# Cost‐Effectiveness of Hepatitis C Virus Case Finding and Treatment in Eastern Europe and Central Asia

**DOI:** 10.1111/liv.70199

**Published:** 2025-07-02

**Authors:** Josephine G. Walker, Irina Tskhomelidze Schumacher, Adam Trickey, Peter Vickerman

**Affiliations:** ^1^ Bristol Medical School University of Bristol Bristol UK; ^2^ Task Force for Global Health Tbilisi Georgia

**Keywords:** blood‐borne infections, Europe, hepatitis C, injecting drug users, modelling, viral infections

## Abstract

**Background and Aims:**

In 2024, < 10% of hepatitis C virus (HCV) cases were treated in Eastern Europe and Central Asia (EECA) and the burden remains high. We aimed to estimate the cost‐effectiveness of treating anyone with HCV (‘treat all’) or targeting people who inject drugs (PWID) in 14 middle‐income EECA countries.

**Methods:**

We gathered costs of screening, confirmatory tests, direct‐acting antiviral (DAA) treatment and monitoring from published country‐specific data, Georgian costs from previous analyses, and UNICEF. We combined decision tree modelling with a dynamic transmission model of HCV calibrated for each EECA country to calculate quality‐adjusted life years (QALYs) gained by 2030 from 100 DAA treatments in 2024, for treat all compared to targeting PWID. We calculated incremental cost‐effectiveness ratios (ICERs, cost per QALY gained) relative to gross domestic product (GDP) per capita.

**Results:**

QALYs gained from 100 treatments ranged from 29‐55 if treat all and 25–90 if targeting PWID. Using country‐level costs, Bulgaria and Russia had ICERs above GDP per capita due to high DAA costs. For other countries, ICERs ranged from 18% to 89% of GDP (treat all) and 4%–89% (PWID). Using lower Georgian costs and UNICEF costs, the treat all ICERs were below 84% and 24% of GDP for all countries, respectively, except Bosnia, while the ICERs when targeting PWID were below 64% and 16% of GDP, respectively.

**Conclusions:**

Strategies that treat all persons with HCV and target PWID are both likely to be cost‐effective in middle‐income EECA countries, particularly with broad access to low‐cost generic treatments such as through UNICEF procurement.

**Abbreviations**
CPI: consumer price index
CrI: credible interval
DAA: direct‐acting antiviral
EECA: Eastern Europe and Central Asia
GDP: gross domestic product
HCV: hepatitis C virus
I$: international dollars
ICER: incremental cost‐effectiveness ratio
LMIC: low or middle‐income country
PCR: polymerase chain reaction
PPP: purchasing power parity
PWID: people who inject drugs
QALY: quality adjusted life year
WHO: World Health Organisation

**Summary**

- Despite global and regional targets for hepatitis C virus (HCV) elimination since 2016, the expansion of access to HCV diagnosis and treatment in Europe has been slow, with only 8% of people living with HCV having been treated by the end of 2022.
- We aimed to inform governments and stakeholders in middle‐income countries on the impact and costs of providing HCV treatment to people who inject drugs (PWID) or treating all. We used a model to project the cost‐effectiveness of HCV diagnosis and treatment in 2024 for 14 middle‐income countries in Eastern Europe and Central Asia.
- Out of 11 countries where published costs were available, treatment was unlikely to be cost‐effective in 8 countries for treating all and 4 countries for PWID; however, when minimal access costs were used, it would be cost‐effective across all 14 countries for both strategies.
- Current costs for diagnostic tests and HCV treatment may still be prohibitive for middle‐income countries that have not yet achieved lower purchase costs or where prevalence is particularly low, but access to recently negotiated minimal costs would make HCV treatment cost‐effective across all the countries examined.

## 1 Introduction

Eastern Europe and Central Asia (EECA) is a region with high hepatitis C virus (HCV) prevalence compared to the global average [1], including countries with a range of income levels [2]. Data from the World Health Organisation (WHO)'s global health observatory for 2022 estimated that the HCV chronic prevalence in EECA ranged from 0.55% in Bosnia and Herzegovina to 3.4% in Ukraine [3]. Among people who inject drugs (PWID), a recent systematic review estimated that the chronic HCV prevalence was 48.6% in Eastern Europe (the highest regional prevalence) and 39.3% in Central Asia, ranging from 12.8% in Czech Republic to 62.9% in Romania [4].

In 2014, highly effective direct‐acting antiviral (DAA) drugs for HCV were developed making HCV easily curable, leading WHO to develop a global strategy to eliminate HCV in 2016. To facilitate the implementation of this elimination strategy, an action plan for the health sector response to viral hepatitis in the WHO European Region was published in 2017 [5, 6]. However, by 2024 only half of EECA countries had developed their own programs or action plans [7]. Of all EECA countries, only Georgia was determined to be ‘on track’ to meet WHO elimination targets in 2021, with Georgia and Slovakia labelled as working towards achieving targets after 2030 in the 2024 update [8, 9].

By the end of 2022, WHO estimated that globally only 36.4% of people living with HCV had been diagnosed and 20% had been treated [10], with only 8% having been treated in the European Region [11]. Of the six European focal countries in the 2024 WHO Global Hepatitis Report, the proportion of HCV cases that have been treated was estimated to be only 4%–5% in Kyrgyzstan, Moldova, Russia and Ukraine, 8% in Uzbekistan and 65% in Georgia [2].

Treatment access will depend on costs of DAA treatments for HCV, and although costs have reduced dramatically since their release in 2014, access to generic drugs and the cost of DAAs varies widely [12, 13, 14]. In EECA, only the lower‐middle income countries (Kyrgyzstan, Tajikistan, Ukraine and Uzbekistan) plus Belarus and Turkmenistan are eligible under voluntary licences to access generic sofosbuvir and daclatasvir. An additional seven countries are eligible to access just daclatasvir (Armenia, Azerbaijan, Bosnia, Bulgaria, Georgia, Kazakhstan and Moldova) [15]. A recent evaluation of the top 20 HCV burden countries found that Russia, with the 10th highest burden globally, faces barriers to achieving elimination due to the high cost of treatment and low public awareness of HCV. Ukraine, with the 20th highest burden, introduced an HCV treatment programme in 2019, but this has been hindered by the war with Russia [16].

To advocate for scaling up programmes towards reaching the WHO elimination targets, EECA governments and stakeholders need data on the potential impact and costs of providing treatment to PWID and the general population. Here, we build on a previous cost‐effectiveness analysis of HCV screening and treatment in Georgia [17, 18], and dynamic modelling of HCV transmission [19], to estimate the cost‐effectiveness of undertaking case‐finding and treatment across the region. We focus our analysis on 14 previously modelled middle‐income countries [19]: Armenia, Azerbaijan, Belarus, Bosnia and Herzegovina, Bulgaria, Georgia, Kazakhstan, Kyrgyzstan, Moldova, Russia, Tajikistan, Turkmenistan, Ukraine and Uzbekistan (noting that Russia and Bulgaria were upgraded to high‐income countries by WHO in mid 2024 [20]).

## 2 Methods

We combined a decision tree model with outputs from an HCV transmission model parameterised for each country [19]. These were combined with cost estimates for HCV screening and treatment for different EECA countries to estimate the cost and cost‐effectiveness of case finding and treatment in PWID and the general population from a healthcare provider's perspective. We calculated an incremental cost‐effectiveness ratio (ICER) in terms of cost per quality adjusted life year (QALY) gained due to 100 treatments in 2024. As willingness‐to‐pay thresholds are not standardised, we present the ICER as a percentage of gross domestic product (GDP) per capita.

### 2.1 Cost Data

We assume the cascade of care starts with an antibody test, such as a rapid diagnostic test, and positive cases require confirmation with a PCR test or Core Antigen test. Patients initiated on treatment will receive treatment with DAAs for 12 weeks with regular clinical monitoring. We did not account for the cost of care for patients with liver disease.

We identified publicly available unit costs or market prices for screening tests, confirmatory tests, treatment monitoring and DAAs for each country by searching Google, Google Scholar and PubMed. Where multiple time points of costs were available, we used the most recent estimate for each country. If country‐specific costs were unavailable, we did not include that country in this part of the analysis. Although different DAAs are available (sofosbuvir/daclatasvir as the most common [15]), in this analysis we used the lowest identified DAA cost per course of treatment identified in each country. Where only DAA costs were available, we used remaining unit costs from the country with the closest purchasing power parity (PPP) adjusted GDP per capita in 2023.

All costs are presented in 2023 international dollars (I$). Values were converted from local currency units to I$ using the PPP conversion factor after costs were inflated to 2023 based on the consumer price index (CPI) in each country as described in Methods S1 and Table S3.

No uncertainty bounds were sampled probabilistically for unit costs, but we undertook sensitivity analyses for all 14 countries using two alternative cost estimates. We firstly used costs from Georgia in 2022 based on a recent in‐depth costing analysis which aimed to capture the full economic costs of screening, diagnosis and treatment, based on financial reimbursement records [18]. Georgian costs from our previous analysis for screening, confirmation and treatment were inflated to I$2.61, $62.57 and $993.90, respectively, with $739.79 of the treatment costs attributable to DAA costs and the rest treatment monitoring [18].

We secondly applied UNICEF procurement costs for screening ($0.80), confirmation ($14.90) and DAAs ($60) to each country [15, 21]. The UNICEF unit costs do not account for staff time or overhead costs, but are just the price of the items (at production) and so represent a lower bound of the costs. To account for this, we added 50% overhead costs to each unit cost [15], resulting in total unit costs of I$1.2, I$22.35 and I$90. For this scenario, we assume a treatment monitoring cost of the lowest country level cost.

### 2.2 Country‐Level Dynamic Model of HCV Transmission

We updated and re‐calibrated a previously described dynamic model of HCV transmission accounting for differential transmission among PWID, HCV treatments undertaken to date, population growth, age demographics and HCV progression [19]. We updated HCV treatment numbers through 2023 and identified updated prevalence estimates from literature as described in the Methods S2, Tables S2 and S2 and Figure S1.

We modelled two alternative intervention scenarios where 100 treatments are either randomly distributed across all infected individuals or are given among PWID alone in 2024. We estimated the future impact of those 100 treatments over 2024 to 2030 in terms of quality‐adjusted life years (QALYs) gained per treatment by applying QALY weights as in previous publications to the annual number of modelled individuals that were susceptible (0.94), infected (0.83), with compensated cirrhosis (0.74), or decompensated cirrhosis (0.66), with a scaling factor for PWID of 0.79 [17]. To calculate the incremental QALYs over 2024 to 2030 to represent the time frame of HCV elimination strategies, these projections were compared to the baseline model which included treatments up to the end of 2023 and no treatments after that. All costs are incurred in 2024 when the treatments are given, while QALYs were discounted 3% per year in the base case as is standard for LMIC economic evaluation [22].

### 2.3 HCV Treatment Decision Tree Model

**FIGURE 1:**
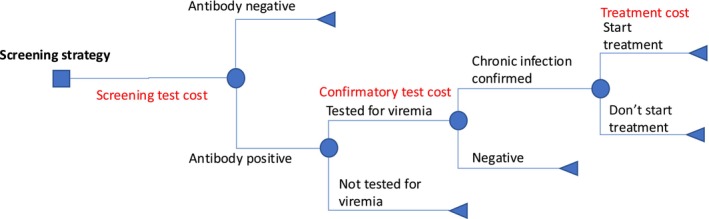
Decision tree structure for screening, confirmation and treatment for hepatitis C virus.

We used a decision tree model (Figure 1) to calculate the total costs related to diagnosing and treating one individual, either a random person or a PWID, based on the HCV transmission model's projections of HCV prevalence in each setting at the beginning of 2024. For simplicity and consistency between settings, we assumed all individuals testing antibody positive receive a confirmatory test (reflex testing), 75% of antibody positive cases are chronically infected (based on what has been observed in Georgia over 2018–2022 [18]), and that 80% of diagnosed individuals initiate treatment as per WHO elimination targets. The cost per treatment is therefore calculated based on the proportion at each end point (antibody negative, viremia negative, treated, or infected but not treated) and the associated cost for each group. Individuals testing antibody negative only have the cost of an antibody screening test, while individuals testing antibody positive, but then viremia negative or not treated, have the cost of an antibody screening test plus RNA test, and lastly individuals that are treated have both test costs and the cost of treatment (including monitoring and DAA costs). We then calculated the total cost per treatment for prevalences from 0.001 to 1 for Georgian and UNICEF costs, to understand how case finding costs vary with screening yield.

### 2.4 Cost‐Effectiveness Analysis

We calculate the incremental cost effectiveness ratio (ICER) as the additional cost of testing and treatment for 100 treatments in 2024 based on the decision tree, divided by the modelled incremental QALYs gained over 2024–2030. We conducted a probabilistic sensitivity analysis in which the ICERs were calculated for each model parameter set and present the mean and 95% credible interval (values between which 95% of modelled values lie). We then present each ICER as the percentage of GDP per capita rather than using a willingness to pay threshold for each setting, as the frequently used threshold of 1–3× GDP per capita is unlikely to represent the true opportunity‐cost threshold [23]. Of the included countries, an official ICER threshold is available only for Bulgaria and Ukraine, both of which were 3× GDP per capita in 2021 [24]. We assume that if an ICER is a lower percentage of GDP then the intervention is more likely to be considered cost‐effective by decision makers in each country.

We conducted sensitivity analysis by changing the discount rate for QALYs to 0%, using a longer time horizon (to 2038), assuming a lower percentage of antibody positive have active infection (50% instead of 75%), and assuming PWID have the same QALY weights as the general population.

## 3 Results

### 3.1 HCV Prevalence

The modelled chronic HCV prevalence in the general population and PWID for each EECA country, and a summary of HCV elimination progress are shown in Table 1. Our model estimates that HCV prevalence reduced or stayed the same in the general population in all settings over 2017–2024. Among PWID, prevalence is projected to have increased in 2 countries (Azerbaijan and Russia) but decreased or remained stable elsewhere.

**TABLE 1:** Country profiles.

Country	Income group	PPP GDP per capita	2017 HCV prevalence among PWID (%, 95% CrI) [19]	2024 HCV prevalence among PWID (%, 95% CrI)	2017 HCV prevalence overall (%, 95% CrI) [19]	2024 HCV prevalence overall (%, 95% CrI)	Total modelled treatments from 2004 to 2023	Estimated population infected in 2024	HCV elimination program status [7]^b^
Armenia	Upper‐middle	23 055	36.0 (26.6, 44.5)	34.9 (23.7, 43.8)	2.4 (1.8, 3.1)	1.8 (1.3, 2.4)	11 496	52 175 (36 250, 76 758)	National program since 2019
Azerbaijan	Upper‐middle	23 686	48.1 (39.5, 56.1)	49.7 (40.7, 58.4)	2.4 (1.8, 3.0)	2.2 (1.7, 2.8)	1890	227 298 (166 578, 297 259)	No action plan found
Belarus	Upper‐middle	30 751	52.8 (40.3, 61.1)	39.9 (31.1, 49.3)	1.1 (0.8, 1.7)	0.8 (0.4, 1.1)	42 000	69 851 (38 078, 109 240)	National action plan since 2020
Bosnia and Herzegovina	Upper‐middle	22 846	41.7 (34.0, 49.1)	3.8 (0.3, 14.1)	0.1 (0.1, 0.1)	0.01 (0.00, 0.04)	5288	317 (19, 1404)	No plan as of 2018 [25]
Bulgaria	Upper‐middle	38 690	50.0 (38.0, 54.6)	48.5 (34.8, 54.9)	1.0 (0.6, 1.5)	0.9 (0.6, 1.2)	13 014	58 451 (38 111, 81 430)	No plan as of 2018 [25]
Georgia	Upper‐middle	24 681	40.5 (28.7, 48.3)	35.3 (23.2, 45.0)	4.6 (4.0, 5.3)	3.3 (2.3, 4.3)	85 458	120 030 (79 935, 165 036)	Elimination program and action plan in place since 2016^a^
Kazakhstan	Upper‐middle	39 332	27.4 (18.9, 30.8)	21.8 (15.0, 27.0)	1.3 (0.8, 1.7)	0.9 (0.5, 1.3)	72 281	172 301 (95 669, 258 846)	National roadmap implemented 2017 [26]
Kyrgyzstan	Lower‐middle	7103	21.6 (19.5, 23.6)	20.1 (17.2, 22.3)	1.5 (0.8, 2.6)	1.3 (0.6, 2.1)	7603	85 057 (41 186, 133 120)	No action plan found^a^
Moldova	Upper‐middle	17 384	41.5 (30.8, 50.4)	33.7 (26.2, 41.5)	2.3 (1.6, 2.8)	1.3 (0.8, 1.9)	33 300	50 910 (27 041, 77 030)	National program since 2017^a^
Russia	Upper‐middle	44 104	47.6 (35.1, 56.6)	48.9 (36.9, 57.1)	2.7 (1.6, 3.6)	2.6 (1.5, 3.4)	213 481	3 678 066 (2 064 339, 5 046 993)	National action plan since 2022 [16]^a^
Tajikistan	Lower‐middle	5082	30.4 (27.8, 32.9)	29.1 (26.6, 31.8)	1.5 (0.7, 2.6)	1.3 (0.6, 2.2)	9483	131 563 (56 011, 221 993)	National program, no date
Turkmenistan	Upper‐middle	17 100	29.9 (24.1, 35.9)	26.5 (21.0, 33.5)	2.0 (0.9, 2.7)	1.4 (0.6, 2.2)	14 966	90 335 (35 479, 142, 134)	No action plan found
Ukraine	Lower‐middle	18 007	37.0 (25.4, 41.3)	32.8 (23.0, 40.0)	2.0 (1.0, 2.7)	1.5 (0.8, 2.2)	68 503	625 951 (306 097, 933 211)	Action plan under development as of 2023^a^
Uzbekistan	Lower‐middle	9725	25.1 (21.4, 29.0)	22.2 (18.7, 26.7)	3.6 (2.3, 4.8)	2.8 (1.7, 4)	106 814	905 073 (527 978, 1 344 259)	Pilot program in 2019 [27]^a^

*Note:* 2017 chronic HCV prevalence estimates are fitted values with 95% credible intervals (CrI) from [19]. 2024 values are updated model projections accounting for treatment through 2023.

^a^ WHO focus country for the viral hepatitis response, as of 2022 reported treatment HCV coverage in these countries are: Georgia, 65%; Uzbekistan, 8%; Russia, 5%; Kyrgyzstan, Moldova and Ukraine, 4% [2].

^b^ The status of HCV elimination and whether programs are in place was compiled from the Coalition for Global Hepatitis Elimination dashboard and National Hepatitis Elimination Profiles [7] and WHO's 2024 Global Hepatitis Report [2].

Primary data‐based estimates were only available for four countries and all other estimates modelled or based on expert opinion, providing little confidence in the data to compare our projections to (Figure S2). While there is clear overlap between other estimates and our confidence bounds in most countries, the model does not align well with other estimates in Bosnia (modelled prevalence particularly low), Belarus and Ukraine.

Across the 14 countries, we estimate that a total of 6 267 377 (95% credible interval [95% CrI]: 3 512 771, 8 888 711) people with HCV remain needing treatment by 2024 (Table 1). Over half of these cases are in Russia (59%), with 14% in Uzbekistan, 10% in Ukraine and 4% in Azerbaijan. The remaining countries each have 3% or less of EECA's burden. Using the upper bound estimate for under‐estimated countries and the lower bound estimate for over‐estimated countries, the estimated number of people with HCV is increased to 6 835 930.

### 3.2 Impact of Treatments

The impact of 100 treatments in 2024 for the random treat all strategy, when projected to 2030, ranged from a median of 29.36 (95% CrI: 26.40, 33.94) QALYs in Georgia to 55.34 QALYs (95% CrI: −32.33, 171.65) in Russia. Instead, targeting PWID leads to more QALYs gained in Bosnia, Kazakhstan, Kyrgyzstan, Tajikistan, Turkmenistan and Uzbekistan, but fewer gained elsewhere. The highest QALY gained for targeting PWID is in Bosnia (90.80, 95% CrI: 35.25, 135.29) and the lowest in Bulgaria (24.60, 95% CrI: 19.19, 33.45). Except for Russia, the 95% CrIs for QALY gains across all countries are positive for treat all or targeting PWID. Total QALYs gained (discounted) are shown in Tables 3 and 4, and undiscounted in Table S2.

### 3.3 Country‐Specific HCV Unit Costs

Our search identified costs for antibody screening, RNA or core antigen confirmation, and treatment from Armenia, Georgia, Kyrgyzstan, Kazakhstan, Ukraine and Uzbekistan (Table 2). To estimate treatment monitoring costs in Kazakhstan and Kyrgyzstan, we found hospital price lists and summed the cost of three clinical visits and nine basic blood tests. DAA costs were unavailable for Armenia, so we assumed the same DAA cost as Georgia due to their proximity, similar GDPs, and same voluntary licence eligibility. Only DAA costs and no other unit costs were available from Moldova ($85.0 per bottle 2019 USD), Tajikistan ($52.0 per bottle 2019 USD) [15] and from Azerbaijan, Bulgaria and Russia ($275.0, $26961.0 and $5831.0 per treatment course, respectively, assumed to be 2019 USD) [14]. Table 2 shows converted costs and which countries were used to fill in the remaining unit costs. For Tajikistan, as CPI is unavailable post‐2016, we could not accurately inflate costs, so assumed all costs are the same as Kyrgyzstan due to the similar 2019 DAA cost and GDP. No cost data were identified for Belarus, Bosnia, or Turkmenistan.

**TABLE 2:** Costs of HCV screening, diagnosis and treatment identified from public domain for selected countries, total cost of treatment, and ICER as % of PPP‐adjusted GDP; costs have been inflated and converted to 2023 international dollars; QALYs gained and GDP shown in Tables 3 and 4.

Country	Screening	Diagnosis	Treatment monitoring	DAA	Cost sources
Armenia	46.83	66.90	434.83	739.79	[28]
Azerbaijan	Armenia costs used			1236.13	[14]
Bulgaria	Kazakhstan costs used			87 536.71	[14]
Georgia	2.61	62.57	254.11	739.79	[18]
Kazakhstan	19.61	22.87	140.52	306.88	[29]
Kyrgyzstan	27.10	137.95	395.78	812.27	[30, 31]
Moldova	Ukraine costs used			1061.86	[30]
Russia	Kazakhstan costs used			23 379.43	[14]
Tajikistan	Kyrgyzstan costs used				[30]
Ukraine	5.77	192.72	2040.94	445.92	[30, 32]
Uzbekistan	14.54	87.83	263.48	690.50	[27, 30]

### 3.4 Cost Per Treatment

In the treat all strategy, the cost per treatment using country‐level costs were generally higher compared to using Georgian costs, with highest costs per treatment being for Bulgaria (I$90469) and Russia (I$24464) due to high DAA costs. In the remaining countries, the cost per treatment ranged from I$1197 in Georgia to I$4701 in Armenia (Table 3). Across all countries, the lowest cost was in Georgia due to higher prevalence there (I$1197 with Georgian costs and $313 with UNICEF costs), with second highest costs in Belarus (I$1529 with Georgian costs and I$466 with UNICEF costs) and much higher costs of I$90251 and I$41258 in Bosnia due to a low estimated prevalence (Table 3).

**TABLE 3:** Cost of treatment, QALYs gained through 2030 from 100 treatments in 2024, and ICER (cost of 100 treatments/incremental QALYs from 100 treatments) as % of PPP‐adjusted GDP in 14 middle‐income countries of Eastern Europe and Central Asia with 3% discounting, in the general population (median and 95% credible interval).

Country	Cost per treatment (2023 International dollars [I$])			QALYs gained^a^	ICER			ICER as % of GDP		
	Country costs	Georgian costs	UNICEF costs	General population	Country costs	Georgian costs	UNICEF costs	Country costs	Georgian costs	UNICEF costs
Armenia	4701 (3717, 5838)	1289 (1234, 1352)	355 (330, 384)	30.8 (27.2, 34.8)	15 239 (12 068, 20 548)	4187 (3697, 4771)	1156 (1010, 1345)	73.3 (58.1, 98.9)	20.2 (17.8, 23.0)	5.6 (4.9, 6.5)
Azerbaijan	4448 (3883, 5212)	1247 (1215, 1289)	336 (322, 356)	34.1 (24.6, 45.8)	13 205 (9584, 18 868)	3663 (2755, 5132)	995 (746, 1380)	61.9 (44.9, 88.4)	17.2 (12.9, 24.0)	4.7 (3.5, 6.5)
Belarus		1529 (1383, 1887)	466 (399, 631)	31.3 (25.0, 42.7)		5004 (3443, 6173)	1532 (1033, 2036)		18.1 (12.4, 22.3)	5.5 (3.7, 7.4)
Bosnia		90 251 (9770, 503 276)	41 258 (4255, 231 154)	34.6 (10.0, 39.1)		247 207 (29 647, 5 090 854)	113 028 (12 911, 2 338 253)		1244.7 (149.3, 25633.3)	569.1 (65.0, 11773.5)
Bulgaria	90 469 (89 806, 92 017)	1465 (1377, 1671)	436 (396, 531)	29.8 (24.3, 35.0)	303 476 (257 411, 374 701)	4944 (4138, 6366)	1457 (1194, 1980)	911.6 (773.3, 1125.6)	14.9 (12.4, 19.1)	4.4 (3.6, 6.0)
Georgia	1197 (1173, 1239)	1197 (1174, 1240)	313 (302, 333)	29.4 (26.4, 33.9)	4074 (3581, 4489)	4075 (3582, 4489)	1070 (931, 1184)	18.3 (16.1, 20.2)	18.3 (16.1, 20.2)	4.8 (4.2, 5.3)
Kazakhstan	3138 (2362, 5306)	1451 (1348, 1740)	430 (383, 563)	32.9 (14.4, 68.1)	9848 (4173, 28 748)	4353 (2092, 11 422)	1296 (614, 3577)	27.8 (11.8, 81.1)	12.3 (5.9, 32.2)	3.7 (1.7, 10.1)
Kyrgyzstan	4277 (3047, 6948)	1372 (1253, 1629)	394 (339, 512)	33.8 (27.0, 44.8)	12 287 (7427, 21 229)	4078 (2921, 5588)	1164 (798, 1689)	191.9 (116.0, 331.6)	63.7 (45.6, 87.3)	18.2 (12.5, 26.4)
Moldova	3963 (3800, 4342)	1342 (1268, 1513)	380 (346, 458)	31.1 (28.0, 36.6)	12 776 (10 885, 14 616)	4352 (3662, 5052)	1227 (1012, 1480)	81.5 (69.5, 93.3)	27.8 (23.4, 32.2)	7.8 (6.5, 9.5)
Russia	24 464 (24 279, 25 195)	1219 (1194, 1316)	323 (312, 368)	55.3 (−32.3, 171.7)	35 173 (−239 411, 605 302)	1766 (−11 880, 29 893)	476 (−3136, 7846)	88.5 (−602.2, 1522.6)	4.4 (−29.9, 75.2)	1.2 (−7.9, 19.7)
Tajikistan	4105 (2991, 7501)	1355 (1248, 1682)	386 (337, 536)	36.1 (22.9, 52.5)	11 499 (6864, 24 400)	3839 (2583, 5967)	1091 (708, 1833)	251.0 (149.8, 532.7)	83.8 (56.4, 130.3)	23.8 (15.5, 40.0)
Turkmenistan		1322 (1244, 1663)	371 (335, 527)	34.0 (29.2, 45.6)		3990 (3009, 5342)	1107 (825, 1664)		23.3 (17.6, 31.2)	6.5 (4.8, 9.7)
Ukraine	3258 (3137, 3744)	1302 (1247, 1521)	361 (336, 462)	37.2 (12.7, 58.6)	8730 (5507, 25 219)	3528 (2189, 10 051)	1017 (591, 2753)	53.8 (33.9, 155.4)	21.7 (13.5, 61.9)	6.3 (3.6, 17.0)
Uzbekistan	1750 (1551, 2189)	1215 (1179, 1294)	321 (304, 358)	39.6 (3.5, 108.7)	4406 (1536, 29 352)	3008 (1087, 21 506)	789 (286, 5613)	50.3 (17.5, 334.9)	34.3 (12.4, 245.3)	9.0 (3.3, 64.0)

^a^ These QALYs include both morbidity gains from reduced disease progression to advanced liver diseases, mortality gains from longer survival due to averted liver disease, and prevention effects from new infections averted.

**FIGURE 2:**
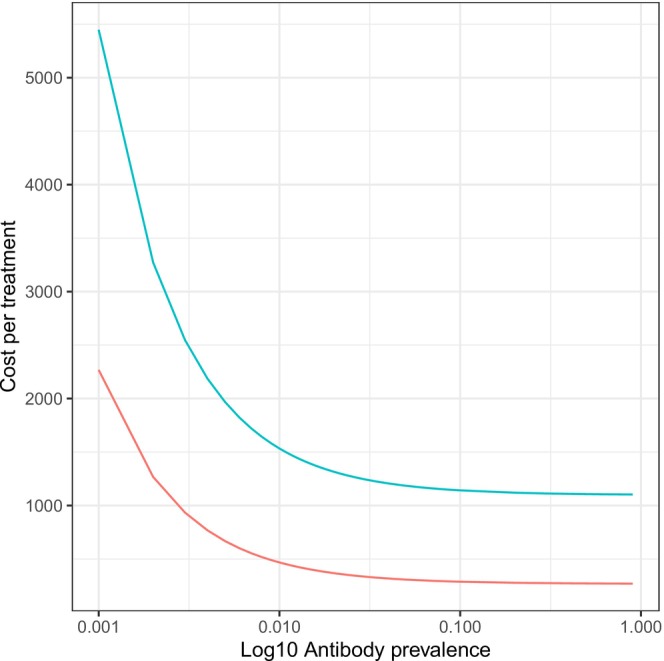
Cost per treatment by antibody prevalence on a log scale using UNICEF costs (red line) and Georgian costs (blue line).

For the strategy targeting PWID, the cost per treatment is lower due to higher prevalence compared to the general population, with country level costs per treatment varying from I$594 in Kazakhstan to I$87764 in Bulgaria (Table 4). Using the Georgian costs, the cost per treatment ranged from I$1104 (for Azerbaijan, Bulgaria and Russia) to I$1290 per treatment (Bosnia) while when using UNICEF costs, the cost per treatment ranged from I$270 to I$356. For the UNICEF or Georgian unit costs, the cost of HCV treatment including the cost of case‐finding reduces as antibody prevalence increases, plateauing when prevalence is greater than 10%. As a result, if the same unit costs are assumed, the total cost per person treated is similar across countries when prevalence is high, such as generally seen in PWID (Figure 2).

**TABLE 4:** Cost of treatment, QALYs gained through 2030 from 100 treatments in 2024, and ICER (cost of 100 treatments/incremental QALYs from 100 treatments) as % of PPP‐adjusted GDP in 14 middle‐income countries of Eastern Europe and Central Asia with 3% discounting, in people who inject drugs (median and 95% credible interval).

Country	Cost per treatment (2023 international dollars [I$])			QALYs gained^a^	ICER			ICER as % of GDP		
	Country costs	Georgian costs	UNICEF costs		Country costs	Georgian costs	UNICEF costs	Country costs	Georgian costs	UNICEF costs
Armenia	1451 (1420, 1533)	1107 (1106, 1112)	272 (271, 274)	27.4 (23.8, 33.5)	5316 (4427, 6061)	4039 (3309, 4659)	992 (814, 1143)	25.6 (21.3, 29.2)	19.4 (15.9, 22.4)	4.8 (3.9, 5.5)
Azerbaijan	1899 (1883, 1926)	1105 (1104, 1106)	271 (270, 271)	28.4 (19.2, 41.8)	6668 (4597, 9882)	3888 (2643, 5761)	952 (648, 1412)	31.2 (21.5, 46.3)	18.2 (12.4, 27.0)	4.5 (3.0, 6.6)
Belarus		1106 (1105, 1109)	272 (271, 273)	28.5 (21.1, 40.0)		3887 (2765, 5245)	955 (679, 1286)		14.0 (10.0, 18.9)	3.4 (2.5, 4.6)
Bosnia		1290 (1121, 2116)	356 (278, 736)	90.8 (35.3, 135.3)		1779 (921, 3215)	528 (248, 912)		9.0 (4.6, 16.2)	2.7 (1.3, 4.6)
Bulgaria	87 764 (87 760, 87 786)	1105 (1104, 1108)	271 (270, 272)	24.6 (19.2, 33.5)	356 782 (262 465, 457 305)	4491 (3313, 5754)	1101 (814, 1410)	1071.8 (788.4, 1373.7)	13.5 (10.0, 17.3)	3.3 (2.5, 4.2)
Georgia	1107 (1105, 1112)	1107 (1105, 1112)	272 (271, 274)	26.7 (23.2, 32.8)	4149 (3386, 4776)	4150 (3386, 4776)	1019 (833, 1171)	18.7 (15.2, 21.5)	18.7 (15.2, 21.5)	4.6 (3.8, 5.3)
Kazakhstan	594 (576, 649)	1113 (1110, 1120)	274 (273, 278)	38.0 (18.6, 74.5)	1601 (807, 3258)	2928 (1497, 5998)	723 (369, 1481)	4.5 (2.3, 9.2)	8.3 (4.2, 16.9)	2.0 (1.0, 4.2)
Kyrgyzstan	1606 (1590, 1635)	1114 (1113, 1117)	275 (274, 277)	38.1 (28.3, 51.5)	4223 (3126, 5738)	2924 (2167, 3941)	722 (535, 9745)	66.0 (48.8, 89.6)	45.7 (33.9, 61.6)	11.3 (8.4, 15.2)
Moldova	3446 (3441, 3452)	1108 (1106, 1111)	272 (271, 274)	28.7 (25.0, 35.6)	11 996 (9682, 13 773)	3857 (3115, 4427)	948 (767, 1087)	76.6 (61. 8, 87.9)	24.6 (19.9, 28.3)	6.1 (4.9, 6.9)
Russia	23 607 (23 601, 23 625)	1105 (1104, 1107)	271 (270, 272)	45.5 (−36.8, 170.4)	34 262 (−133 796, 175 478)	1603 (−6265, 8209)	393 (−1537, 2011)	86.2 (−336.6, 441.4)	4.0 (−15.8, 20.7)	1.0 (−3.9, 5.1)
Tajikistan	1554 (1544, 1565)	1109 (1108, 1110)	273 (272, 273)	38.1 (23.5, 54.3)	4091 (2880, 6603)	2916 (2046, 4721)	718 (504, 1c161)	89.3 (62.9, 144.2)	63.7 (44.7, 103.1)	15.7 (11.0, 25.4)
Turkmenistan		1111 (1108, 1114)	273 (272, 275)	37.3 (30.2, 50.1)		2982 (2216, 3680)	735 (545, 908)		17.4 (13.0, 21.5)	4.3 (3.2, 5.3)
Ukraine	2830 (2826, 2840)	1108 (1106, 1112)	272 (272, 274)	35.9 (6.5, 58.5)	7885 (4845, 43 340)	3087 (1897, 16 971)	758 (467, 4172)	48.6 (29.9, 267.0)	19.0 (11.7, 104.6)	4.7 (2.9, 25.7)
Uzbekistan	1184 (1169, 1198)	1113 (1110, 1116)	275 (273, 276)	43.7 (6.1, 109.3)	2708 (1089, 22 694)	2547 (1020, 21 338)	628 (252, 5265)	30.9 (12.4, 258.9)	29.1 (11.6, 243.4)	7.2 (2.9, 60.1)

^a^ These QALYs include both morbidity gains from reduced disease progression to advanced liver diseases, mortality gains from longer survival due to averted liver disease, and prevention effects from new infections averted.

### 3.5 Cost Per QALY

For the treat all strategy (Table 3), the ICER ranged from I$4074/QALY in Georgia to I$303476/QALY in Bulgaria using country‐level costs. The ICER as percent of GDP was lowest in Georgia (18%) followed by Kazakhstan (28%) with it being 9‐times the GDP per capita in Bulgaria. Remaining countries had ICERs between 50%–89% of GDP per capita, except Tajikistan (2.5‐times higher) and Kyrgyzstan (1.9‐times higher). Using Georgian costs and UNICEF costs reduced the ICER for all countries to below 84% and 24% of the GDP, respectively, with the exception of Bosnia where the ICERs were 12.4× and 5.7× GDP per capita for Georgian costs and UNICEF costs, respectively.

The ICERs from targeted treatment for PWID are shown in Table 4, with country‐level costs resulting in ICERs from I$1600/QALY in Kazakhstan up to I$356782/QALY in Bulgaria. Russia had the most uncertainty; the lower bound had negative incremental QALYs (Figure S2). Armenia, Azerbaijan, Georgia, Kazakhstan, Ukraine and Uzbekistan all had ICERs lower than 50% of GDP per capita, while Kyrgyzstan, Moldova, Russia and Tajikistan had ICERs between 65% and 90% of GDP per capita. For treating PWID, Bulgaria's ICER was approximately 10 times the GDP per capita, though when lower costs are used this reduces to 13.5% (Georgian costs) or 3.3% (UNICEF costs) of the GDP, indicating that a reduction in DAA costs compared to the list price would have a drastic impact on the cost‐effectiveness of HCV case finding and treatment there. When using Georgian costs, all countries have median ICERs < 50% GDP per capita except Tajikistan (64%). When using UNICEF costs, all countries have ICERs < 16% of GDP per capita, with 10 countries < 5%. These ICERs are very likely to be considered as highly cost‐effective.

Recent conflicting empirical estimates of willingness to pay thresholds for each country have been made and reported in terms of cost per QALY (or DALY) as a percent of GDP per capita, and these generally come out below 1× GDP per capita (Table S4) [23, 33]. In our analysis, using UNICEF costs would put both treat all and PWID targeted treatment below those estimated thresholds for all countries. Similarly, when using Georgia costs for all countries, then both treatment strategies would fall below these thresholds except treat all in Bosnia and either strategy in Tajikistan. In contrast, when country costs are used, the treat all strategy is only cost‐effective in Belarus, Georgia and Turkmenistan, while the PWID targeted strategy is only cost‐effective in Armenia, Bosnia, Belarus, Georgia, Kazakhstan and Kyrgyzstan (Table S4).

### 3.6 Sensitivity Analysis

Sensitivity analysis results are shown in Figures S3–S5. Extending the time horizon made the biggest difference to reducing the ICER in all settings, while the other changes made minimal difference to the interpretation of the ICERs regardless of which cost inputs were used.

## 4 Discussion

Despite the 14 middle‐income countries of EECA being heterogeneous in terms of viral hepatitis burden and progress towards elimination, with only seven having action plans [2, 34], there is still potential for impact from HCV treatment. We found between 24 and 90 QALYs gained by 2030 for every 100 DAA treatments in 2024, whether these are given randomly or targeted to PWID. We also identified that as antibody prevalence increases, the cost of treatment including case finding decreases and then plateaus, with the cost of treatment only increasing significantly when the antibody prevalence is below 10%. Therefore, due to high antibody prevalence among PWID, the cost per treatment was similar across all countries when the same testing and treatment costs were used.

Using a treat all strategy or targeting PWID are both likely to be cost‐effective in most of the 14 middle‐income countries modelled, although reductions in cost to match Georgian costs or UNICEF costs with 50% overheads improve cost‐effectiveness. Decision makers in each country will need to make choices based on their own guidelines for priority setting and for those where the cost is still prohibitively high, explore access to lower cost diagnostic tools and DAAs and limit overhead costs.

To our knowledge, no previous studies have used dynamic models to evaluate the cost‐effectiveness of hepatitis C diagnosis and treatment strategies across different populations or cost estimates in our target region of EECA. Previously, individual country‐level analyses have been conducted for many countries since the introduction of DAAs, including in EECA (Georgia [17, 35], Ukraine (focused on PWID [36]) and Russia [37]). Furthermore, a general model for evaluating the cost‐effectiveness of DAAs is available that covers 38 countries, including Georgia, Kyrgyzstan, Ukraine and Uzbekistan, but this uses a static model with limited cost inputs [38]. Generally, the published studies find that HCV treatment will be cost‐effective if DAA costs are low enough, which this study complements with more detailed costing and comparison of how total treatment costs change with prevalence.

### 4.1 Strengths and Limitations

A strength of this study is the use of a previously validated HCV transmission model to project the QALYs gained by treatments. The assumed number of treatments implemented prior to 2024 may affect the number of infections that are projected to be averted by each treatment, and therefore the total QALY impact. However, this represents the current state in each country. The short time horizon to 2030 will underestimate the treatment benefits, which will accrue over time, and so our cost‐effectiveness estimates are conservative, as we saw in sensitivity analysis when using a time horizon to 2038.

We had to make a number of simplifying assumptions for this evaluation across multiple countries. We did not account for differences in treatment effectiveness or the proportion of chronically infected individuals by country. In the dynamic model, we did not have enough updated prevalence estimates to re‐fit the model, and as a result our prevalence estimates may not be accurate. We assume that overall prevalence or prevalence among PWID represents the screening yield which would be found.

We filled gaps in cost data by drawing on data from other countries, and costs in each country may have changed since the published estimates we identified, particularly as the cost of DAAs is rapidly changing. Furthermore, the cost estimates for treatment monitoring represent different resource usage for each estimate, with these costs likely to be the most uncertain. By using the same unit costs across each country for sensitivity analysis, we explored the relative impact of differences in epidemiology vs. differences in input costs for each country on their cost‐effectiveness estimates.

### 4.2 Implications

To reach elimination targets as set by WHO, rapid increases in diagnosis and treatment initiation will be needed in the EECA and the rest of the European region, where it's estimated only 8% of people with hepatitis C have been treated [11]. A survey of 16 EECA countries in 2016 found that HCV programs covered 0.15% of people needing treatment [39]. Since then, only half of the EECA countries have initiated action plans or elimination programs on a national level, despite the elimination target of 2030. There is severe inequity in access to HCV treatment, with high‐income countries more likely to have treated a larger proportion of HCV cases [40]. This pattern may be driven by the small number of countries that have made good progress towards HCV elimination, with disparities not only in strategic plans related to viral hepatitis and resources to implement the plans, but in the total burden of infection due to differing prevalences in each country.

In EECA and other high burden countries globally, wide scale screening at the population level as well as targeting PWID will be needed to achieve elimination targets. In Russia and Bulgaria, where DAA costs are high, this will require access to cheaper generic drugs and/or price negotiation with manufacturers as has been achieved in Ukraine [32]. Testing strategies can build on approaches proven effective in Georgia and other countries which have decentralised and integrated HCV testing and linkage to care, including integration in PWID intervention settings and prisons for targeted screening, and emergency departments and antenatal care for general population screening [41]. Such strategies have been found to be cost‐effective even in high income countries with higher baseline costs, including screening and treatment of pregnant women in the US [42], and among PWID in the UK [43]. Furthermore, a previous modelling analysis found that when indirect costs of productivity losses are included, HCV elimination programs are likely to be cost saving to society [44].

HCV diagnosis pathways and treatment should be accessible to everyone, and our results show that treatment for all can be cost‐effective across the different settings that we modelled. Initially, the high cost of new DAAs was a barrier for many countries in the region. After voluntary licences became available to some countries this allowed for the local production or importation of generic versions of these DAAs. However, some countries in the EECA region were not included in these voluntary licences, leaving them with high costs [15]. Market reports show generally declining prices for diagnosis and treatment, and recent agreement of a global ceiling price for sofosbuvir and daclatasvir at $60 for 12 weeks of treatment will make treatment more affordable for many [15]. However, the true costs to a country or individual in procuring and accessing HCV diagnostic tests and drugs will depend on the policy landscape, including voluntary licensing access, and ability to make bulk purchases. The global ceiling price, for example, requires a minimum purchase of 2500 or 3500 packs from the WHO pre‐qualified generic manufacturers [15]. Since the introduction of DAAs, there has been enormous variation in DAA pricing at a country level, with little correlation with a country's economic status [13, 14]. However, our findings show that in EECA, the cost of diagnosis and of HCV treatment with DAAs can be low enough such that even in low‐income countries and countries with lower prevalence, treatment would be considered cost‐effective whether treating the general population or focusing on PWID.

## Author Contributions

**Josephine G. Walker:** conceptualisation, methodology, software, data curation, writing – original draft preparation, visualisation and formal analysis. **Irina Tskhomelidze Schumacher:** investigation, data curation, validation and writing – review and editing. **Adam Trickey:** conceptualisation, methodology, software and writing – review and editing. **Peter Vickerman:** conceptualisation, supervision and writing – review and editing.

## Ethics Statement

No ethical approval was required for this study, which was a modelling study using secondary data.

## Conflicts of Interest

This study was funded by Gilead Sciences through an unrestricted investigator‐sponsored research grant to P.V. and J.G.W.

## Supporting information


Data S1. CHEERS Checklist

Data S2. Supplementary Methods, Figures, and Tables

## Data Availability

All data included in manuscript and Supporting Information S1 and S2, code available on request from authors.
