# Supplementary material for: Cost‐Effectiveness of Hepatitis C Virus Case Finding and Treatment in Eastern Europe and Central Asia
Source: Liver Int. 2025 Jul 2;45(8):e70199. doi: 10.1111/liv.70199 (PMC12217427; doi:10.1111/liv.70199)
Supplement: Supplementary file 2 — Data S2. Supplementary Methods, Figures, and Tables [file LIV-45-0-s001.docx]

# Supplementary Material

Cost-effectiveness of Hepatitis C virus case finding and treatment in Eastern Europe and Central Asia

Josephine G. Walker^1^, Irina Tskhomelidze Schumacher^2^, Adam Trickey^1^^, Peter Vickerman^1^^

**Affiliations**

1. Bristol Medical School, University of Bristol
2. Task Force for Global Health, Tbilisi, Georgia

^ Equal contribution

**Correspondence:** josephine.walker@bristol.ac.uk

Table of Contents

[Supplementary Methods 2](#_Toc193799017)

[Supplementary Figures 3](#_Toc193799018)

[Supplementary Tables 8](#_Toc193799019)

[References 12](#_Toc193799020)

## Supplementary Methods

**Cost Inflation**

Values were converted from local currency units to international dollars (I$) using the purchasing-power parity (PPP) conversion factor after costs were inflated to 2023 based on the consumer price index (CPI) in each country. If CPI was only available to 2021 (Russia) or 2022 (Uzbekistan) we assumed the annual rate of inflation was the same as in the last reported year. Where original cost data was in another currency, such as United States dollars, this was first inflated to 2023 and converted to local currency units before conversion to I$ based on PPP. UNICEF costs presented in $ were assumed to be US costs and therefore correspond to I$. GDP per capita was based on current I$ PPP-based GDP per capita for 2023, except for Turkmenistan, where the latest PPP-based GDP per capita was for 2019^1^.

**Dynamic model updates**

To update the model, we searched Google Scholar for publications from 2016 onwards that reported hepatitis C virus (HCV) prevalence in each country (search terms “hepatitis C prevalence [country name]”). We identified new country-specific chronic HCV prevalence estimates from several sources, with 1-4 new estimates being identified for each country over 2016-2022, including modelled Polaris estimates from 2020 and WHO global health observatory estimates from 2022^2,3^ (Supplementary Table 2).

We collated treatment numbers from 2015-2022 for 17 countries from the WHO global health observatory data^4^, and from two publications; over 2016-2020 for 7 countries in central Europe^5^ and 4 countries in Eastern Europe^6^. For Georgia, we used programmatic data on numbers treated in the elimination program over 2015-2023^7^. Where possible, we reconciled treatment numbers across multiple sources, and assumed the same number were treated in 2023 as 2022, allowing us to update the treatment numbers in each country model for 2015-2023 (Supplementary Table 1). Only one country (Azerbaijan) had no updated treatment data available, so we assumed the same number of treatments per year as used in the previously published version of the model^8^.

We re-calibrated the model using the same methods as previously described^8^ to identify 100 parameter sets (model runs) per country. In some cases, runs failed to fit so the total number of accepted parameter sets was between 77-100 for each country, with 7/14 countries having 100 model fits. We did not re-calibrate the model to new HCV prevalence estimates but compared our updated model projections (accounting for additional treatments) to these new estimates to determine how well the model fit them (Supplementary Figure 1).

## Supplementary Figures

**Supplementary Figure 1:** Chronic HCV prevalence overall by country as modelled from 2015 through 2030 (lines, mean value, ribbon, 95% credible interval) with points and error bars (where available) showing published estimates since 2016 of chronic prevalence in each setting (sources in Supplementary Table 3). Survey (data) estimates shown in filled circles, other modelled estimates as X, expert opinion as triangle, and WHO global health observatory as +. Boxes in top right of each figure show the number (out of 100) of model runs used.

**Supplementary Figure 2:** Incremental QALYs that accumulate by 2030 from 100 additional treatments in 2024 applied to the whole population (treat all, red, solid line) or targeted for people who inject drugs (PWID, blue, dashed line), in each country. Lines and ribbons show mean estimate and 95% credible interval across model runs. The area under the curve represents the total undiscounted incremental QALY benefit of the 100 treatments by 2030.

**Supplementary Figure 3:** Sensitivity analysis showing changes to the ICER under a longer time horizon (LongHorizon), chronic prevalence of 50% instead of 75% (LowChronic), 0% discount rate (NoDiscount), and no difference in QALY weights for PWID compared to the general population (PWIDweight). ICERs are using country level costs (I$ per QALY gained from treatment), dashed lines show GDP per capita. Dashed line shows GDP per capita, dots show median ICERs and 95% credible intervals. PWID – treatments for PWID, TA – treat all strategy.

**Supplementary Figure 4**: Georgian cost sensitivity analysis showing changes to the ICER under a longer time horizon (LongHorizon), chronic prevalence of 50% instead of 75% (LowChronic), 0% discount rate (NoDiscount), and no difference in QALY weights for PWID compared to the general population (PWIDweight). ICERs are using Georgian costs (I$ per QALY gained from treatment). Dashed line shows GDP per capita, dots show median ICERs and 95% credible intervals. PWID – treatments for PWID, TA – treat all strategy.

**Supplementary Figure 5**: UNICEF cost sensitivity analysis showing changes to the ICER under a longer time horizon (LongHorizon), chronic prevalence of 50% instead of 75% (LowChronic), 0% discount rate (NoDiscount), and no difference in QALY weights for PWID compared to the general population (PWIDweight). ICERs are using UNICEF costs (I$ per QALY gained from treatment). Dashed line shows GDP per capita, dots show median ICERs and 95% credible intervals. PWID – treatments for PWID, TA – treat all strategy.

## Supplementary Tables

**Supplementary Table 1: Updated treatment numbers for 2015-2023 (numbers prior to 2015 as in** ^8^**)**

| **Country** | **2015** | **2016** | **2017** | **2018** | **2019** | **2020** | **2021** | **2022** | **2023** | **Source comments** |
| --- | --- | --- | --- | --- | --- | --- | --- | --- | --- | --- |
| Armenia | 1366 | 1366 | 1366 | 1366 | 1366 | 1366 | 1366 | 934 | 1000 | WHO data is high compared to other estimates which indicate program started in 2020, but there seems to be a decline in prevalence so used WHO treatment estimates.^4,9^ |
| Azerbaijan | 210 | 210 | 210 | 210 | 210 | 210 | 210 | 210 | 210 | No new data found ^8^ |
| Belarus | 0 | 0 | 0 | 5000 | 9000 | 7000 | 7000 | 7000 | 7000 | Account for reimbursed and privately purchased estimates ^6^ |
| Bosnia | 635 | 635 | 635 | 635 | 635 | 635 | 635 | 443 | 400 | Divide total 2015-2021 evenly ^4^ |
| Bulgaria | 611 | 720 | 1325 | 1230 | 1000 | 1000 | 1000 | 1000 | 1000 | ^5^ 2016-2020 |
| Georgia | 5932 | 21650 | 14784 | 10196 | 11961 | 8275 | 3834 | 4254 | 4572 | ^7^ accessed programmatic data by year |
| Kazakhstan | 5450 | 5450 | 5450 | 5450 | 5450 | 5450 | 5450 | 7731 | 7000 | WHO data split evenly over 2015-2021 ^4^ |
| Kyrgyzstan | 965 | 965 | 965 | 965 | 965 | 965 | 965 | 448 | 400 | WHO data split evenly over 2015-2022 ^4^ |
| Moldova | 300 | 3900 | 3900 | 3900 | 3900 | 3900 | 4500 | 4500 | 4500 | ^6,10^ |
| Russia | 17586 | 17586 | 17586 | 17586 | 17586 | 17586 | 17586 | 28079 | 28000 | Divide total 2015-2021 evenly ^4^ |
| Tajikistan | 1055 | 1055 | 1055 | 1055 | 1055 | 1055 | 1055 | 1048 | 1050 | Divide total 2015-2021 evenly ^4^ |
| Turkmenistan | 1648 | 1648 | 1648 | 1648 | 1648 | 1648 | 1648 | 1730 | 1700 | Divide total 2015-2021 evenly ^4^ |
| Ukraine | 6089 | 6089 | 6089 | 6089 | 6089 | 6089 | 6089 | 12780 | 12000 | Divide total 2015-2021 evenly ^4^ |
| Uzbekistan | 12402 | 12402 | 12402 | 12402 | 12402 | 12402 | 12402 | 10000 | 10000 | Divide total 2015-2021 evenly ^4^ |

**Supplementary Table 2:** Updated chronic prevalence estimates identified from literature as shown as points in Supplementary Figure 1.

| **Country** | **Prevalence estimate** | **Lower bound** | **Upper bound** | **Year** | **Source** |
| --- | --- | --- | --- | --- | --- |
| Armenia | 2.2 | 1.9 | 3.1 | 2020 | Polaris 2020 modelled ^3^ |
| Azerbaijan | 1.9 | 1.2 | 2.3 | 2020 |  |
| Bulgaria | 1.2 | 0.6 | 1.9 | 2020 |  |
| Georgia | 2.4 | 2.4 | 2.8 | 2020 |  |
| Kazakhstan | 1.9 | 1.6 | 2.2 | 2020 |  |
| Kyrgyzstan | 2.6 | 2.2 | 4.1 | 2020 |  |
| Russia | 2.9 | 1.8 | 3.4 | 2020 |  |
| Tajikistan | 2.7 | 2 | 3.1 | 2020 |  |
| Ukraine | 3.1 | 2.5 | 4 | 2020 |  |
| Uzbekistan | 3 | 2.4 | 3.6 | 2020 |  |
| Armenia | 0.7 |  |  | 2022 | WHO global health observatory ^2^ |
| Belarus | 2.12 |  |  | 2022 |  |
| Bosnia | 0.55 |  |  | 2022 |  |
| Bulgaria | 0.92 |  |  | 2022 |  |
| Georgia | 2.26 |  |  | 2022 |  |
| Kazakhstan | 2.02 |  |  | 2022 |  |
| Kyrgyzstan | 2.51 |  |  | 2022 |  |
| Moldova | 2.12 |  |  | 2022 |  |
| Russia | 1.85 |  |  | 2022 |  |
| Tajikistan | 2.57 |  |  | 2022 |  |
| Turkmenistan | 2.53 |  |  | 2022 |  |
| Ukraine | 3.4 |  |  | 2022 |  |
| Uzbekistan | 3.02 |  |  | 2022 |  |
| Bulgaria | 1.11 | 0.83 | 1.6 | 2019 | ^11^ |
| Armenia | 0.7 | 0.4 | 1 | 2021 | ^12^ |
| Bulgaria | 0.9 | 0.2 | 4.2 | 2018 | ^13^ |
| Georgia | 1.8 | 1.3 | 2.4 | 2021 | ^14^ |
| Russia | 1.1 | 0.8 | 1.4 | 2016 | ^15^ |
| Bulgaria | 1.1 |  |  | 2020 | ^5^ |

**Supplementary Table 3: Data from World Bank used for currency conversion** ^1^**; LCU, local currency units; CPI, consumer price index; GDP, gross domestic product; PPP, purchasing power parity**

| **Country** | **GDP per capita, PPP (current international $) in 2023 - updated date 13/11/2024** | **CPI 2016** | **CPI 2018** | **CPI 2019** | **CPI 2021** | **CPI 2022** | **CPI 2023** | **2023 LCU per USD (Official exchange rate LCU per I$, period average)** | **2023 LCU per I$ (PPP conversion factor GDP)** |
| --- | --- | --- | --- | --- | --- | --- | --- | --- | --- |
| Armenia | 23,055 | 123.01 | 127.34 | 129.18 | 140.13 | 152.24 | 155.26 | 392.48 | 148.09 |
| Azerbaijan | 23,686 | 132.38 | 152.90 | 156.89 | 171.94 | 195.76 | 212.96 | 1.70 | 0.51 |
| Belarus | 30,751 | 432.91 | 481.39 | 508.34 | 587.30 | 676.63 | 710.46 | 3.01 | 0.77 |
| Bosnia and Herzegovina | 22,846 |  |  |  |  |  |  | 1.81 | 0.67 |
| Bulgaria | 38,690 | 105.76 | 110.97 | 114.42 | 120.17 | 138.58 | 151.67 | 1.81 | 0.74 |
| Georgia | 24,681 | 117.11 | 127.43 | 133.61 | 154.01 | 172.34 | 176.62 | 2.63 | 0.87 |
| Kazakhstan | 39,332 | 157.56 | 179.72 | 189.30 | 218.27 | 251.07 | 288.04 | 456.17 | 153.01 |
| Kyrgyz Republic | 7,103 | 146.93 | 153.93 | 155.68 | 185.23 | 211.02 | 233.71 | 87.86 | 24.36 |
| Moldova | 17,384 | 144.36 | 158.53 | 166.20 | 181.26 | 233.35 | 264.66 | 18.16 | 6.95 |
| Russian Federation | 44,104 | 162.20 | 173.02 | 180.75 | 199.37 |  |  | 85.16 | 26.67 |
| Tajikistan | 5,082 | 148.57 |  |  |  |  |  | 10.84 | 2.54 |
| Turkmenistan | 17,100* |  |  |  |  |  |  |  | 1.45 |
| Ukraine | 18,007 | 205.61 | 261.07 | 281.66 | 316.45 | 380.32 | 429.19 | 36.57 | 10.52 |
| Uzbekistan | 9,725 | 185.13 | 247.77 | 283.76 | 355.02 | 395.66 | ^ | 11734.83 | 3010.59 |

*GDP per capita for Turkmenistan is from 2019; ^Assume 10% increase compared to 2022

**Supplementary Table 4: Cost-effectiveness of treatment strategies compared to estimated willingness to pay (WTP) thresholds as percent of GDP per capita.**

| Country | WTP threshold A (cost/QALY) ^16^ | WTP threshold B (cost/DALY) ^17^ | General population^ | PWID^ |
| --- | --- | --- | --- | --- |
| Armenia | 96% | 27% - 41% | C^A^; G^AB^; U^AB^ | C^AB^; G^AB^; U^AB^ |
| Azerbaijan | 33% | 24-36% | G^AB^; U^AB^ | C^A^; G^AB^; U^AB^ |
| Belarus | 49% | 54-87% | *G^AB^; U^AB^ | *G^AB^; U^AB^ |
| Bosnia | 79% | NA | *None | *G^A^; U^A^ |
| Bulgaria | 60% | 58-85% | G^AB^; U^AB^ | G^AB^; U^AB^ |
| Georgia | 52% | 20-27% | C^AB^; G^AB^; U^AB^ | C^AB^; G^AB^; U^AB^ |
| Kazakhstan | 23% | 36-55% | C^B^; G^AB^; U^AB^ | C^AB^; G^AB^; U^AB^ |
| Kyrgyzstan | 31% | 58-88% | U^AB^ | G^B^; U^AB^ |
| Moldova | 43% | 85-127% | C^B^; G^AB^; U^AB^ | C^B^; G^AB^; U^AB^ |
| Russia | 47% | 51-82% | G^AB^; U^AB^ | G^AB^; U^AB^ |
| Tajikistan | 31% | 35-48% | U^AB^ | U^AB^ |
| Turkmenistan | 50% | 25-33% | *G^AB^; U^AB^ | *G^AB^; U^AB^ |
| Ukraine | 46% | 50-77% | G^AB^; U^AB^ | C^B^; G^AB^; U^AB^ |
| Uzbekistan | 38% | 46-67% | G^AB^; U^AB^ | C^AB^; G^AB^; U^AB^ |

^Treat all and PWID columns show which scenario is cost-effective below threshold; C, country-level costs cost-effective; G, Georgia costs cost-effective; U, UNICEF costs cost-effective; superscript A and B indicate which WTP thresholds it is below, using the lower bound from WTP threshold B. *Belarus, Bosnia, and Turkmenistan did not have country-level costs available

## References

1 World Bank Group. World Bank Open Data. World Bank Open Data. https://data.worldbank.org (accessed Sept 2, 2024).

2 World Health Organization. The Global Health Observatory Indicator ‘Hepatitis - prevalence of chronic hepatitis among the general population’. 2024. https://www.who.int/data/gho/data/indicators/indicator-details/GHO/hepatitis---prevalence-of-chronic-hepatitis-among-the-general-population (accessed Dec 9, 2024).

3 Blach S, Terrault NA, Tacke F, *et al.* Global change in hepatitis C virus prevalence and cascade of care between 2015 and 2020: a modelling study. *Lancet Gastroenterol Hepatol* 2022; **7**: 396–415.

4 World Health Organization. The Global Health Observatory Indicator ‘Hepatitis - number of persons initiated hepatitis C treatment, latest year and cumulative over a period of years’. 2024. https://www.who.int/data/gho/data/indicators/indicator-details/GHO/hepatitis---prevalence-of-chronic-hepatitis-among-the-general-population (accessed Dec 9, 2024).

5 Flisiak R, Zarębska-Michaluk D, Frankova S, *et al.* Is elimination of HCV in 2030 realistic in Central Europe. *Liver Int* 2021; **41**: 56–60.

6 Isakov V, Tsyrkunov V, Nikityuk D. Is elimination of hepatitis C virus realistic by 2030: Eastern Europe. *Liver Int* 2021; **41**: 50–5.

7 Tohme RA, Shadaker S, Adamia E, *et al.* Progress Toward the Elimination of Hepatitis B and Hepatitis C in the Country of Georgia, April 2015–April 2024. *MMWR Morb Mortal Wkly Rep* 2024; **73**: 660–6.

8 Trickey A, Fraser H, Lim AG, *et al.* Modelling the potential prevention benefits of a treat‐all hepatitis C treatment strategy at global, regional and country levels: A modelling study. *J Viral Hepat* 2019; **26**: 1388–403.

9 Armenian health authorities adopt policy of eliminating viral hepatitis by 2022-2030 strategy. Armenpress. 2022; published online July 28. https://armenpress.am/en/article/1089152 (accessed Dec 13, 2024).

10 Tcaciuc E, Holban T, Plăcintă G, Vasiliţa S, Ardeleanu D, Olaru-Stăvilă C. hepatitei cronice virale C în Republica Moldova. .

11 Thomadakis C, Gountas I, Duffell E, *et al.* Prevalence of chronic HCV infection in EU/EEA countries in 2019 using multiparameter evidence synthesis. *Lancet Reg Health - Eur* 2023; **36**: 100792.

12 Demirchyan A, Mozalevskis A, Sahakyan S, *et al.* Seroprevalence of Hepatitis C Virus and Factors Associated with It in Armenia, 2021. *Viruses* 2024; **16**: 1446.

13 Sperle I, Nielsen S, Gassowski M, *et al.* Prevalence of hepatitis C in the adult population of Bulgaria: a pilot study. *BMC Res Notes* 2020; **13**: 326.

14 Gamkrelidze A, Shadaker S, Tsereteli M, *et al.* Nationwide Hepatitis C Serosurvey and Progress Towards Hepatitis C Virus Elimination in the Country of Georgia, 2021. *J Infect Dis* 2023; **228**: 684–93.

15 Isakov V, Nikityuk D. Elimination of HCV in Russia: Barriers and Perspective. *Viruses* 2022; **14**: 790.

16 Pichon-Riviere A, Drummond M, Palacios A, Garcia-Marti S, Augustovski F. Determining the efficiency path to universal health coverage: cost-effectiveness thresholds for 174 countries based on growth in life expectancy and health expenditures. *Lancet Glob Health* 2023; **11**: e833–42.

17 Ochalek J, Lomas J, Claxton K. Estimating health opportunity costs in low-income and middle-income countries: a novel approach and evidence from cross-country data. *BMJ Glob Health* 2018; **3**: e000964.
